# Supplementary material for: Asynchrony of Gambierdiscus spp. Abundance and Toxicity in the U.S. Virgin Islands: Implications for Monitoring and Management of Ciguatera
Source: Toxins (Basel). 2021 Jun 10;13(6):413. doi: 10.3390/toxins13060413 (PMC8230442; doi:10.3390/toxins13060413)
Supplement: Supplementary file 1 [file toxins-13-00413-s001.zip › toxins-1228149 supplementary for conversion.pdf]

Supplementary Materials: Asynchrony of *Gambierdiscus* spp. Abundance and Toxicity in the U.S. Virgin Islands: Implications for Monitoring and Management of Ciguatera

Justin D. Liefer, Mindy L. Richlen, Tyler B. Smith, Jennifer L. DeBose, Yixiao Xu, Donald M. Anderson and Alison Robertson

Table S1. Pearson's correlations for environmental variables and the first two principal components (PC1 and PC2) from a principal components analysis of environmental data collected in this study. All data except nutrient concentrations were one week means. Values in bold are significant based on the broken stick criterion of Peres-Neto et al. 2003 [1].

Variable	PC1	PC2
Wind Speed (m s^{-1})	-0.448	**0.690**
Wind Direction (degrees)	**0.855**	0.028
Precipitation (mm)	0.492	0.608
Benthic Temperature (°C)	**0.766**	0.187
Ammonium (µM)	0.067	-0.445
Silicate (µM)	-0.083	0.416
Phosphate (µM)	-0.008	-0.274
Nitrate + Nitrite (µM)	-0.230	0.410
Proportion of Variance	22.8%	18.69%
Cumulative Proportion of Variance	22.8%	41.49%

Table S2. Results of an ANOSIM test (Primer-E) comparing macroalgal composition at each study site. For the values of the ANOSIM test statistic R shown, a value of 1 corresponds to complete multivariate dissimilarity while 0 indicates sites are essentially identical. Asterisk* indicates significant results, $p < 0.001$.

Field Site → Field Site ↓	Coculus Rock	Black Point	Flat Cay
**Seahorse Shoal**	0.824 *	0.935 *	0.47 *
**Flat Cay**	0.501 *	0.539 *	
**Black Point**	0.14		

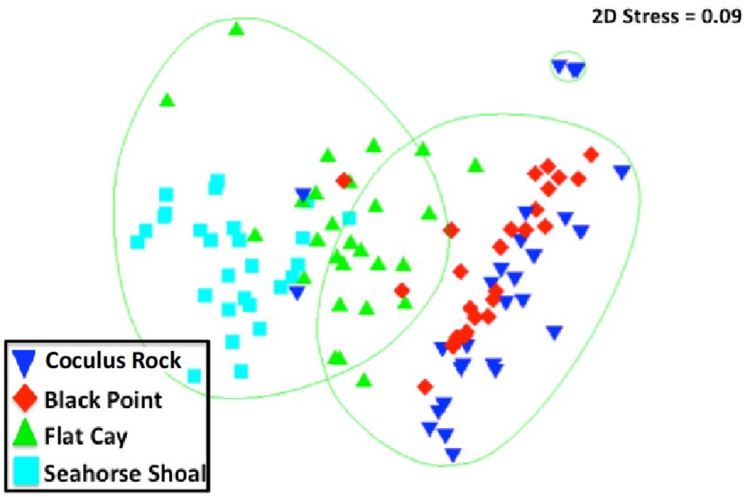

Figure S1. A non-metric multidimensional scaling plot (nMDS) and results of ANOSIM test for benthic macroalgal composition at each site. nMDS visualization is based on Bray – Curtis similarity of sites. The circles on the nMDS plot indicate 77% similarity based on hierarchical cluster analysis.

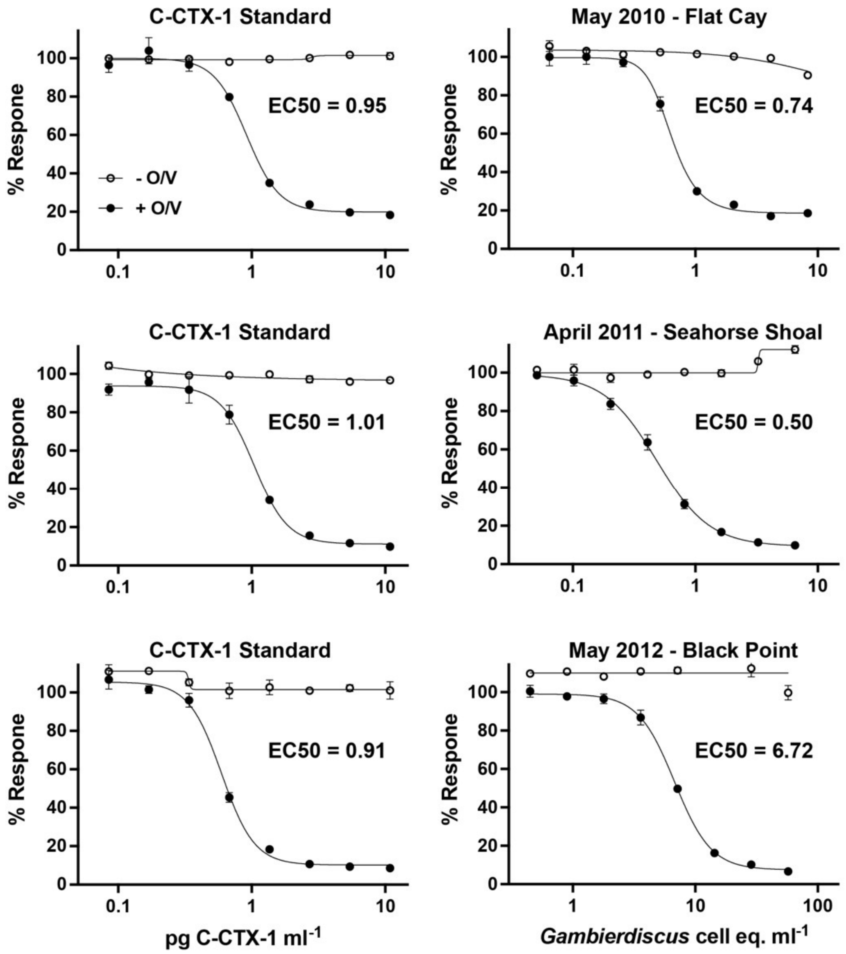

Figure S2. Examples of N2a assay dose-response curves for purified C-CTX-1 standards (left column panels) and field epiphyte samples (right column panels). The field samples shown are representative of the shape and direction of the dose-response for all quantified samples. The standard assay to the left of each field sample assay was performed concurrently with that field sample assay, with both assays utilizing the same population of N2a cells for initial seeding of assay plates. In addition to receiving a dose of standard or sample, N2a cells were also pre-treated with a solution of ouabain/veratridine (+O/V, closed circles) or phosphate-buffered saline (-O/V, open circles). The % response shown is based the production of a formazan product (measured by absorbance at 570nm) through metabolic reduction of MTT, a proxy of metabolic activity and cell viability, and was normalized to the response of -O/V and +O/V control cells. The dose concentrations reflect exposure conditions for N2a cells within assay wells. Field sample doses are expressed as *Gambierdiscus* cell equivalents based on the volume of extract used for dosing and the *Gambierdiscus* cell abundance measured in each sample prior to pelleting and extraction. The dose concentration corresponding to a 50% normalized response in +O/V cells (EC50) was calculated from a four-parameter sigmoidal curve fit; the EC50 measured in a standard assay and concurrent sample assays was used to determine the toxin concentration (as C-CTX-1 equivalents) in each sample. Error bars indicate the standard deviation of triplicate measurements and in some cases are obscured by the symbol.

1. Peres-Neto, P.R.; Jackson, D.A.; Somers, K.M. Giving meaningful interpretation to ordination axes: Assessing loading significance in Principal Component Analysis *Ecology* **2003**, *84*, 2347-2363, doi:https://doi.org/10.1890/00-0634.
